# Quality and Trace Element Profile of Tunisian Olive Oils Obtained from Plants Irrigated with Treated Wastewater

**DOI:** 10.1100/2012/535781

**Published:** 2012-05-01

**Authors:** Cinzia Benincasa, Mariem Gharsallaoui, Enzo Perri, Caterina Briccoli Bati, Mohamed Ayadi, Moncen Khlif, Slimane Gabsi

**Affiliations:** ^1^Centro di Ricerca per l'Olivicoltura e l'Industria Olearia, CRA, via Li Rocchi 111, 87036 Rende, Italy; ^2^Olive Tree Institute, University of Sfax, Route de l'aeroport Km 1.5, BP 1087, 3000 Sfax, Tunisia; ^3^National School of Engineering, University of Gabes, Rue Omar Ibn El Khattab 6029, Tunisia

## Abstract

In the present work the use of treated wastewater (TWW) to irrigate olive plants was monitored. This type of water is characterized by high salinity and retains a substantial amount of trace elements, organic and metallic compounds that can be transferred into the soil and into the plants and fruits. In order to evaluate the impact of TWW on the overall quality of the oils, the time of contact of the olives with the soil has been taken into account. Multi-element data were obtained using ICP-MS. Nineteen elements (Li, B, Na, Mg, Al, K, Ca, Sc, Cr, Mn, Fe, Co, Ni, Cu, Zn, Sr, Mo, Ba and La) were submitted for statistical analysis. Using analysis of variance, linear discriminant analysis and principal component analysis it was possible to differentiate between oils produced from different batches of olives whose plants received different types of water. Also, the results showed that there was correlation between the elemental and mineral composition of the water used to irrigate the olive plots and the elemental and mineral composition of the oils.

## 1. Introduction

Tunisia is a very important country in the olive oil producing world, the largest, African exporter and fourth worldwide after Spain, Italy and Greece with an annual average export over 10,000 metric tonnes [1]. The olive tree (*Olea europaea* L.) is present practically in every region of the country up to the border of the southern desert. Tunisia belongs to the Middle East and North Africa (MENA), which is considered one of the driest regions in the world [2]; in fact, only 30% of the cultivated area in the region is irrigated but produces about 75% of the total agricultural production. Alternative water resources are therefore needed to satisfy further increases in demand. Tunisia launched a national water reuse program in the early 1980s to increase usable water resources. Most municipal wastewater is from domestic sources and receives secondary biological treatment. In 2003, 187 million m^3^ (78%) of the 240 million m^3^ of wastewater collected in Tunisia received treatment. About 30–43% of the treated wastewater (TWW) was used for agricultural and landscape irrigation [3]. Reusing wastewater for irrigation is viewed as a way to increase water resources, provide supplemental nutrients, and protect coastal areas, water resources, and sensitive receiving bodies. Reclaimed water is used on 8000 ha to irrigate industrial (cotton, tobacco, sugarbeet, etc.) and fodder crops (alfalfa, sorghum, etc.), cereals, vineyards, citrus, and other fruit trees (olives, peaches, pears, apples, pomegranates, etc.). Regulations allow the use of secondary-treated effluent on all crops except vegetables, whether eaten raw or cooked [4]. As it is demonstrated that the olive trees respond favourably and efficiently to the irrigation management [5–7] and as the water resources in Tunisia are limited, the use of nonconventional water can be a good alternative. However, due to the particular characteristics of the water and potential health risks associated with their use in agriculture, it is important that the effects of irrigation with TWW are objectively evaluated. The primary and secondary wastewater treatments improve distinctly the water quality as the treated wastewater still retains a substantial amount of organic, and metallic compounds [8, 9] such as carbon, nitrogen, phosphorous, and potassium which have a favourable effect on the growth of certain crops [10]. Moreover, the reuse of the TWW can also have important consequences for the irrigated soils as the TWW can change their characteristics and quality due to the process of salinization and pollution by some mineral, organic, and bacteriological materials [10]. Several authors have studied the impact of TWW on the quality of the soils [11–19]. The possibility of transfer metals and trace elements by using TWW into the soil and from the soil to the plant is not well studied. The most commonly used techniques for the determination of metals in oil samples are inductively coupled plasma atomic emission spectrometry (ICP-AES) and atomic absorption spectrometry (AAS) [20]. Fats and oils are particularly difficult to analyze for their trace metal contents since some of them are present at very low concentration levels. For this reason inductively coupled plasma mass spectrometry (ICP-MS) is considered an interesting tool because of its well-known high sensitivity and because it allows a simultaneous quantitative determination of multielements in such a complex matrix [21]. However, since ICP-MS analysis faces many drawbacks, sample needs to be treated in order to eliminate the organic matrix that causes the extinction of the plasma. The aim of this study was, therefore, to determine the impact of TWW used to irrigate olive trees on the quality of the olive oils produced. Since farmers in Tunisia collect fallen olives, especially if the quantity that has dropped is consistent and mix them with olives harvested by hand directly from the tree, it is essential to control also the quality of oils extracted from olives fallen on field irrigated with TWW [22]. Hence, this paper proposes a quantitative analysis of elements in olive oils from Chemlali cultivar produced in Tunisia by means of ICP-MS.

## 2. Experimental

### 2.1. Olive Sampling and Oil Extraction

The olive samples utilized in this study were collected from experimental plots located in the region of Sfax in central eastern Tunisia in the experimental station of El Hajeb. These experimental plots are characterized by sandy soil, and the use of the TWW is for the irrigation of annual crops in insertion with the olive trees. If olive trees are in insertion with alfalfa they receive water at an annual rate of 10,000 m^3^ per ha by means of a continuous drip irrigation system. During the experimental years, irrigation was performed every month (continuous irrigation) with the exception of the harvest period that occurred on December and January. If olive trees are in insertion with oats, they receive water at an annual rate of 5,000 m^3^ per ha by means of a continuous drip irrigation system that was performed from April to May and from October to December (alternate irrigation). Twenty-two-year-old trees spaced 24∗24 m were used in block design with three different irrigation systems: (a) olive trees intercropped with alfalfa receiving continuous irrigation with TWW since 2001; (b) olive trees intercropped with oats receiving an alternating irrigation with TWW since 2001; (c) olive trees irrigated with conventional water (CW), (d) olive trees not irrigated (control plants). For each irrigation system, fallen olives were picked from under the trees. The residence time of the olives on the ground is from a few days to several months, and, normally, their moisture percentage content reflects their residence time under the olive trees and then in contact with the soil. Therefore, once collected, olives were sorted according to their moisture percentage content and called as follows: first fall 50.73% (±5.31%), second fall 33.45% (±5.15%), third fall 14.89% (±2.62%) and forth fall 4.22% (±0.72%). The moisture percentage of the olives collected directly from the plant resulted to be 44.22% (±7.45%). For each irrigation system, 3 kg of olives were milled by means of a laboratory scale hammer mill. After 20 minute of malaxation, the oil was separated by centrifugation.

### 2.2. Chemical Characteristics of Treated Wastewater and Conventional Water [23]

In order to remove the biodegradable matters of TWW, biological processes are performed by the action of aerobic microorganisms that, in the presence of oxygen, are able to metabolize the organic matter producing more microorganisms and inorganic end products such as CO_2_, NH_3_, and H_2_O. Conventional water (CW) characteristics are quite different from those of TWW. Calculated values of pH resulted in 7.60 and 7.95 for CW and TWW, respectively, and, as they fall within the range of 6 to 9, their use can be employed in agriculture [24]. The electrical conductivity for TWW was 6.30 dS/m while 4.60 dS/m for CW indicating, respectively, a high and moderate level of salinity [25]. The concentration of NH_4_^+^, NO_3_^−^, and *P*_total_ was high (37.9, 15.9, and 10.3 mg/mL compared to 2.24, 1.11 and 0.8 mg/mL of CW, resp.). The concentrations of Na^+^ and Cl^−^ in TWW were 470 mg/mL and 1999 mg/mL while, in CW, was 355 mg/mL and 1580 mg/mL. In both TWW and CW, chloride concentration was higher than the threshold reported by Chartzoulakis in the guidelines for olive irrigation [26, 27]. The concentrations of Ca^2+^ (96 mg/mL) and Mg^2+^ (84 mg/mL) were almost half compared with those present in CW. The concentrations of Zn^2+^ (0.42 mg/mL) and Mn^2+^ (0.5 mg/mL) were four and five times higher than in CW. Although the values of COD and BOD were very high in TWW (73 mg/mL and 22 mg/mL) compared to the values in CW, both biological and chemical oxygen demands were below the Tunisian thresholds for water reuse (30 and 90 mg/mL, resp.).

### 2.3. Physicochemical Analysis of Olive Oils

The olive oils were analysed for their most important physicochemical parameters, that is, free acidity, peroxide index, UV absorption characteristics at 232 and 270 nm, and fatty acid methyl esters according to the official methods of European Union [28].

### 2.4. Total Phenols Analysis

Total phenols content were determined as described in our previous paper [29]. In brief, 2.5 g of olive oil were dissolved in hexane and extraction with a solution of methanol and water was performed. The phenolic fraction was determined by mean of Folin-Ciocalteu reagent and quantitation achieved by external calibration curve (*r* ^2^ = 0.996) made with caffeic acid purchased from Sigma Aldrich.

### 2.5. Fatty Acid Methyl Esters (FAMEs) Analysis

Fatty acid methyl esters (FAMEs) analysis were carried out as described in our previous paper [30]. FAMEs were analyzed by gas chromatography, and peaks were identified by comparing their retention times with those of authentic reference compounds. The fatty acid composition was expressed as relative percentages of each fatty acid calculated considering the internal normalization of the chromatographic peak area.

### 2.6. ICP-MS Analysis

#### 2.6.1. Materials and Apparatus for ICP-MS Analysis

The ultrapure HNO_3_ (normaton ultrapure, VWR prolabo) used in this work was acquired by analytical-reagent grade certified for the impurities. Single- and multielement standards (Certipur, Merk, Darmstadt, Germany) were also analytical-reagent grade. Aqueous solutions were prepared using ultrapure water, with a resistivity of 18.2 Mcm, obtained from a Milli-Q plus system (Millipore, Saint Quentin Yvelines, France). All glassware were decontaminated with nitric acid (2%, v/v) for at least two hours, rinsed with ultrapure water, and dried. The experimental work was carried out using a Milestone MLS-1200 MEGA oven system for the microwave digestion. The determination of the elements of interest was carried out by means of an Agilent 7500e ICP-MS instrument (Agilent Technologies, Santa Clara, USA) where oil samples were introduced by means of a quartz nebulizer. The ICP torch was a standard torch equipped with platinum injector. As the performance of the ICP-MS instrument strongly depends on the operating conditions [31], a solution containing Rh, Mg, Pb, Ba and Ce (10 g/L) was used to optimize the instrument in terms of sensitivity, resolution, and mass calibration. The ^140^Ce^16^O^+^/^140^Ce^+^ ratio was used to check the level of oxide ions in the plasma that could interfere in the determination of some elements; also, instrumental parameters such as RF power and carrier gas flow were optimized and the level of doubly charged ion monitored by means of the signal ^137^Ba^2+^/^137^Ba^+^. ICP-MS analysis was performed following the operating program and parameters as follows: plasma power: 1150 W; nebuliser: glass concentric type; carrier gas flow rate: 1.05 l/min; oxide ratios: < 3% (CeO/Ce); double charged species: <2% (Ba^2+^/Ba^+^); sample uptake rate: 1.2 mL/min; coolant argon flow: 17 l/min; sweeps/reading: 45; readings/replicate: 3; replicates: 3; dwell time: 55 ms; scan mode: peak hopping; isotopes monitored: ^7^Li^+^, ^9^Be^+^, ^10^B^+^, ^23^Na^+^, ^25^Mg^+^, ^27^Al^+^, ^29^Si^+^, ^31^P^+^, ^34^S^+^, ^39 ^K^+^, ^43^Ca^+^, ^45^Sc^+^, ^49^Ti^+^, ^51^V^+^, ^53^Cr^+^, ^55^Mn^+^, ^57^Fe^+^, ^59^Co^+^, ^62^Ni^+^, ^65^Cu^+^, ^66^Zn^+^, ^74^Ge^+^, ^75^As^+^, ^82^Se^+^, ^88^Sr^+^, ^89^Y^+^, ^90^Zr^+^, ^93^Nb^+^, ^95^Mo^+^, ^111^Cd^+^, ^121^Sb^+^, ^139^La^+^, ^140^Ce^+^, ^141^Pr^+^, ^145^Nd^+^, ^147^Sm^+^, ^151^Eu^+^, ^158^Gd^+^, ^159^Tb^+^, ^163^Dy^+^, ^165^Ho^+^,^169^Tm^+^, ^173^Yb^+^, ^175^Lu^+^, ^181^Re^+^, ^205^Tl^+^, ^208^Pb^+^, ^232^Th^+^.

#### 2.6.2. Sample Preparation

Sample preparation was performed as reported in Benincasa et al. [21]. Briefly, each olive oil sample was homogenized by vigorous shaking, and 0.5 g was weighed directly into the digestion vessel where nitric acid was added.

Olive oil samples were digested and analysed three times in order to check the sensitivity and reproducibility of the digestion procedure. The operating program for the microwave digestion system consisted of 6 steps, and the total time of the digestion procedure lasted 31 minutes. After cooling at room temperature, all the digestion liquors were quantitatively transferred into volumetric flask and diluted to volume (30 mL) with ultrapure water. An analytical batch contained 3 procedural blanks and two procedural blanks spiked with a standard solution containing 48 elements. A mid-range calibration standard was measured at the end of each analytical run, for quality control purposes, that is, to assess instrumental drift throughout the run. Limits of quantitations (LOQs) were defined as 10 times the standard deviation of the signal from reagent blanks, after correction for sample weight and dilution. All the elements that were below this value were not accepted for statistical analysis.

#### 2.6.3. Calibration Procedure

In order to quantify the elements in the oils, external calibration curves were build on five different concentrations. Standard solutions were prepared by diluting a multielement solution of Ce, Dy, Er, Eu, Gd, Ho, La, Lu, Nd, Pr, Sm, Sc, Tb, Th, Tm, Y, and Yb at 10 mg/mL; a multielement solution of Ag, Al, B, Ba, Bi, Ca, Cd, Co, Cr, Cu, Fe, K, Li, Mg, Mn, Mo, Na, Ni, Pb, Sr, Tl, and Zn at 100 mg/mL; a multielement solution of Au, Ge, Pt, Sn, Ti, and Zr at 10 mg/mL; a solution of Si, S, and P at 1000 mg/mL. The concentration range for the elements were between 0.01–100 mg/mL and 0.2–2000 mg/mL. Two spike solutions were used as a recovery test.

### 2.7. Statistical Analysis

All statistical treatment was performed by STATGRAPHICS Plus Version 5.1 (Statistical Graphics Corporation, Professional Edition—Copyright 1994–2001).

The statistical approach that has been chosen to analyse the set of data obtained by ICP-MS was principal component analysis (PCA) and linear discriminant analysis (LDA).

Also, in order to check possible differences between the oils, two-way analysis of variance (ANOVA) was performed considering, as main factors, the irrigation regimes and the type of irrigation water. Moreover, to evaluate significant differences between averages, Tukey test was performed on the oil quality parameters. Differences were considered statistically significant for *P* ≥ 0.01 (capital letters) and *P* ≥ 0.05 (small letters). The values obtained for free acidity and FAMEs were statistically analyzed after arcsine transformation in order to meet assumptions for ANOVA. However, the results are presented in their original scale of measurement and reported as the averages of three repetitions (*n* = 3).

## 3. Result and Discussions

### 3.1. Physicochemical Analysis of Olive Oils

The most important quality parameters of the oils analysed in this study are listed in Table 1. In general, oils obtained from olives whose plants were irrigated with TWW were found to be “lampanti” characterized by high values of free acidity and low values of polyphenols. This result could be explained by considering that the quality of edible oils and fats is effected by their concentrations of trace metals [32, 33]. Traces of metals in edible oils are known to have an effect on the rate of oil oxidation, decreasing the shelf life of commercial products [34]. Apart from causing premature rancidity, these oxidation processes may generate peroxides, aldehydes, ketones, acids, epoxides, and other compounds that may cause effects in the digestive system and also react with other food components (proteins and pigments), sensitising the action of some carcinogens [35].

### 3.2. Free Fatty Acid

Analysis of variance (ANOVA) applied to the values of free acidity has clearly made a distinction between the oils under investigation. Considering as main factors the harvesting mode, oils obtained from olives that remained in contact with the soil for a short period gave a value of free acidity comprised in a range between 1.52 and 3.27%, while oils obtained from olives that stayed for longer periods gave values up to 6.42%. Oils obtained from olives harvested directly from the plants gave a value of 0.95%.

Considering the second factor being the water regime, oils obtained from olives whose plants were irrigated with TWW were found to be of poor quality and characterized by high values of free acidity. However, we must point out that oils produced from olives harvested directly from the plant (control plants) gave a value of free acidity of 6.19% in an alternate irrigation regime with TWW and 6.80% in a continuous one; 0.72% in the rain-fed regime and 0.58% in the conventional water one (Table 1).

### 3.3. Polyphenols

Considering the first factor analysed being the harvesting mode, it is highly evident that the most abundant content of polyphenols is found in the oils produced from olives harvested directly from the plants. This value decreases significantly increasing the contact of the fruits with the soil. In fact, the polyphenols content of oils produced by processing the fruits collected from the ground in the last period of the harvest was very low (12.32 mg/kg). Considering the second factor being the water regime; the content of polyphenols found in the oils obtained from olives belonging to the rainfed regime resulted to be statistically different from all the other values (50.87 mg/kg). This value decreases significantly within the water regime, in fact, oils coming from plant irrigated with TWW gave values of 26.36 and 21.29 mg/kg for the continuous and alternate system, respectively, while 37.28 mg/kg for the conventional one (Table 1).

### 3.4. Fatty Acid Methyl Esters (FAMEs)

In general, the fatty acid content is typical of Tunisian oils [36, 37]. However, the oils under analysis were dominated by palmitic acid (C16: 0), stearic acid (C18: 0), oleic acid (C18: 1), and linoleic acid (C18: 2) (Table 1). In this study, the observed values do not show a particular pattern that can explain the permanence of the olives with the soil and the incidence of the different water irrigation regimes on the quality of the oils obtained. In fact, the fatty acids of an olive oil are not dependent on the processing and harvesting of the olives but rather depend on genetic factors [38, 39].

### 3.5. Quality Control and Quality Assurance Data of ICP-MS Analysis

Initially, 48 elements were investigated but only 19 were submitted for statistical analysis. The criteria utilized to select those elements were as follows: recovery data were accepted if results were in the range of 70–120%, with 80% within 80–110% and for CRM values, within 20%. The results must be not below the limit of detection (LOD). The replicate agreement was considered acceptable if the value of the RSD was minor of 10%.  Table 2 details the LOD values and the percentage recovery of a known amount of analytes spiked for all the elements submitted for statistical analysis. Results from spikes and recovery experiments at levels of 30, 80, and 300 ng/mL were in the range 92–104%, for almost all the elements. Li, Ca, and Ge gave a value of 74, 80, and 87%, respectively, whereas Ba and Tl gave a value of 111 and 120%, respectively. Furthermore, it can be seen from Table 2, the values of LODs which were in the range of 0.000–0.051 mg/kg, for almost all the elements. Fe, Ca, Al and Mg gave a value of 0.294, 0.587, 0.953, and 5.118 mg/kg respectively. Values of LOQs were in the range of 0.000–0.400 for almost all the elements except for Fe, K, Ca, Al and Mg that gave a value of 0.980, 1.068, 1.956, 3.178, and 17.061 mg/kg respectively. The relative standard deviations of the elements were less than 2% for all the elements.

### 3.6. Statistical Analysis of Multielement Data

**Figure 1:**
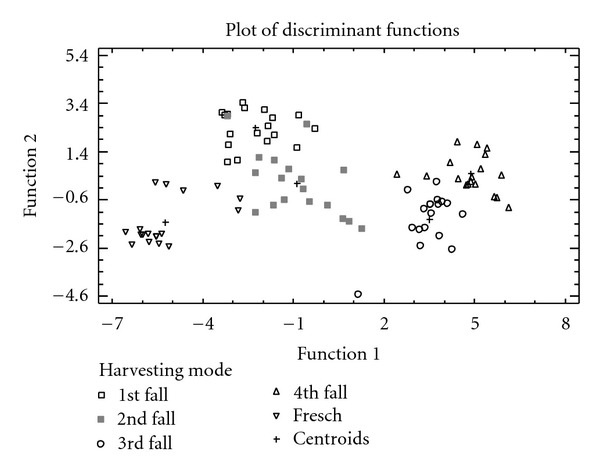
LDA plot for 90 olive oil samples (30 samples repeated three times) based on the concentration of 19 elements and using as input a priori five groups, that is, those corresponding to the olives harvested directly from the plants, called fresh, and those that have fallen naturally and stayed in contact with the soil irrigated with TWW. The moisture percentage of the olives were as follows: 50.73% (±5.31%) called 1st fall; 33.45% (±5.15%) called 2nd fall; 14.89% (±2.62%) called 3rd fall; 4.22% (±0.72%) called 4th fall.

At first, the trace elements profile and the minerals content present in the oils obtained from olives harvested from the ground irrigated with TWW have been monitored (Table 3). In order to develop a model to discriminate among the 5 levels of type of olive (see *Olive Sampling and Oil Extraction*), 90 cases (30 olive oil samples three time replicated) were used and 19 predictor variables (Li, B, Na, Mg, Al, K, Ca, Sc, Cr, Mn, Fe, Co, Ni, Cu, Zn, Sr, Mo, Ba, and La) were entered. The LDA plot resulting is showed in Figure 1, while Table 4 lists the summing up of the analyses of the discriminating functions.

The scores of the first two functions produced from LDA showed a separation into 3 groups: a first group is represented only by oils produced from olives hand picked from the plant (control plants) independently of irrigation regime, a second group is formed by oils produced from olives that stayed in contact with the ground for a short period and having a moisture containing comprised between 50.73% and 33.45% (1st and 2nd fall), and a third group formed by oils produced from olives that stayed in contact with the ground for very long time and having a moisture containing comprised in a range of 14.89% and 4.22% (3rd and 4th fall).

In particular, the control plants produced oils with a lower concentration of trace elements and minerals. These oils are lying in the lower left portion of the plot function characterized by negative values.

The oils produced from olives collected from the ground after a short permanence are characterized by higher values of Na, K, Ni, La, Sr, B, and Mo, while those produced from olives collected from the ground after a longer period are characterized by higher values of Li, Cu, Fe, Mn, and Sc (Table 4).

In order to verify the reliability of the model, the method has been tested using known samples as unknown variables. In particular, a set of 5 samples, composed of one sample for each category of olives, was randomly removed for five times and the model was recalculated. Amongst the 90 observations used to fit the model, 78 or 86.67% were correctly classified.

Accordingly, by the PCA applied to the concentration of the 19 elements of each single sample, 5 principal components have been extracted, having Eigenvalues greater than or equal to 1.0, and together they account for 71% of the variability in the original data. The elements that mainly contributed to the separation of the groups are Al, Sr, Ba, and Mg on PC1 and Na, K, and Cr on PC 2.

**Figure 2:**
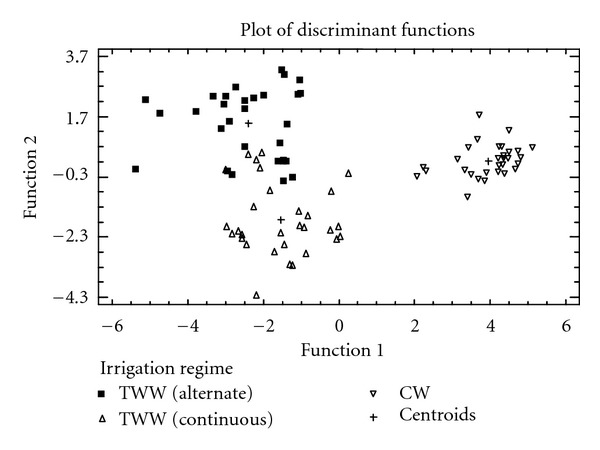
LDA plot for 90 olive oil samples (30 samples repeated three times) based on the concentration of 19 elements and using as input a priori three groups, that is, those corresponding to the irrigation regimes: alternate and continuous using TWW and CW.

In a second time, LDA was applied to the data set considering as input a priori the irrigation regimes. The bidimensional plot of the first two functions shows a clean separation into 2 groups: oils produced from olives whose plants were irrigated with TWW (in a continuous and alternate regime) and oils obtained from olives whose plants were irrigated with CW (Figure 2).

Amongst the 90 observations used to fit the model, 79 or 88% were correctly classified. When PCA was applied, 7 components have been extracted having Eigenvalues greater than or equal to 1.0, and together they account for 74% of the variability in the original data. The elements that mainly contributed to the separation of the groups are mainly Sc, Mn, Li, Cu, Fe, La, Ni, K, and Na on PC1 and Mg, Ba, Al, Ca, Sr and B on PC 2.

## 4. Conclusions

The development of rapid and accurate analytical methods for the determination of metal concentrations in edible oils and fats is still a challenge in terms of quality control analysis, owing to the low concentration levels of some elements and the difficulties that arise due to the characteristics of the matrix [21, 40]. The metals analysed in this study, particularly Fe, Cu, Ca, Mg, Ni, and Mn, are known to increase the rate of oil oxidation [41, 42].

Statistical analysis clearly showed how the way to harvest the olives strongly influences the quality of oils produced [32]. Oils obtained from olives harvested from the ground were found in fact to be of poor quality. This data is not surprising considering that these olives are source of fermentation processes that lead to high values of free acidity and very low values of phenolic compounds in the oil. Moreover, if we consider the contact of the olives with the moist soil, surely the produced oil will be marked by the negative attribute of mould and ground. All the ICP-MS results and statistical evaluations performed have showed that oils produced from olives harvested from the ground have a richer trace elemental and mineral profile than oils produced from olives picked directly from the plant. In particular, olive trees irrigated with treated wastewater produced oils even richer in trace elements and minerals.

The European Community sets limit only for PB in edible oils, which is 0.1 mg/kg (European Commission 2006) [43]; for the other elements, no limits are set and their concentrations could be a concern for consumers.

Therefore, this study has pointed out that the irrigation with TWW adversely affects the quality of the oils. In fact, the olive oils were found to be “lampanti” characterized by high values of free acidity and very low values of polyphenols. Although the availability of wastewater is a very interesting alternative for urban agriculture, the health risks associated with this practice can constitute a real obstacle to the development of this activity.

## Figures and Tables

**Table 1:** Quality parameters of the olive oils under investigation.

Harvesting mode based on the olives percentage humidity	Palmitic acid (%)		Stearic acid (%)			Oleic acid (%)			Linoleic acid (%)			Free acidity (% oleic acid)		Polyphenols (mg kg^−1^)		K232			K270	
From the tree	19.72	A	1.24	a	A	50.53	d	B	24.97	a	A	0.95	E	70.01	A	2.35	c	B	0.13	D
First fall	19.93	A	1.04	c	CD	51.66	c	B	24.17	ab	AB	1.52	D	42.80	B	2.42	c	B	0.17	C
Second fall	19.98	A	1.11	b	B	51.49	c	B	23.99	abc	AB	3.27	C	25.92	C	2.70	b	A	0.20	B
Third fall	19.09	B	0.92	d	D	53.16	b	A	23.82	bc	AB	5.70	B	18.72	D	2.92	a	A	0.24	A
Forth fall	18.70	B	1.12	b	BC	54.27	a	A	23.04	c	B	6.42	A	12.32	E	2.89	ab	A	0.23	A

Water regimes																				
TWW (alternate)	19.35	B	1.39		A	50.71		B	24.90		AB	6.19	B	21.29	D	2.83	a	AB	0.26	A
TWW (continuous)	17.40	C	0.94		C	57.32		A	21.48		C	6.80	A	26.36	C	2.32	c	C	0.21	B
Not irrigated	19.67	B	0.71		D	51.49		B	25.58		A	0.72	C	50.87	A	2.63	b	B	0.15	C
CW	21.52	A	1.30		B	49.37		C	24.03		B	0.58	C	37.28	B	2.84	a	A	0.16	C

Means, for each factor, in the same column followed by the same letter do not differ according to Tukey's test (capital letters *P* < 0.01; small letters *P* < 0.05). Values are the mean of three replications.

**Table 2:** Limit of quantitations (LOQs), limit of detections (LODs), percentage recovery of spike solutions analysed by ICP-MS.

	Spike 1 (rec.)	Spike 2 (rec.)	LOD	LOQ
	%	%	mg kg^−1^	mg kg^−1^
^7^Li	84	74	0,005	0,016
^10^B	96	89	0,051	0,170
^23^Na	95	90	0,104	0,347
^25^Mg	109	95	5,118	17,061
^27^Al	96	98	0,953	3,178
^39 ^K	100	97	0,319	1,063
^43^Ca	80	74	0,587	1,956
^45^Sc	103	96	0,000	0,000
^49^Ti	104	103	0,025	0,084
^53^Cr	104	103	0,000	0,000
^55^Mn	104	102	0,012	0,039
^57^Fe	92	90	0,294	0,980
^59^Co	105	104	0,005	0,015
^62^Ni	105	105	0,011	0,038
^65^Cu	104	104	0,000	0,000
^74^Ge	87	69	0,000	0,000
^88^Sr	106	103	0,006	0,019
^90^Zr	101	101	0,039	0,130
^95^Mo	103	101	0,028	0,092
^111^Cd	92	92	0,000	0,000
^118^Sn	99	93	0,000	0,000
^137^Ba	111	108	0,007	0,023
^139^La	103	102	0,017	0,056
^140^Ce	102	101	0,000	0,000
^141^Pr	103	102	0,000	0,000
^145^Nd	103	104	0,000	0,000
^147^Sm	104	103	0,000	0,000
^151^Eu	103	102	0,000	0,000
^158^Gd	104	103	0,000	0,000
^159^Tb	102	101	0,000	0,000
^163^Dy	104	102	0,000	0,000
^165^Ho	101	100	0,000	0,000
^166^Er	104	103	0,000	0,000
^169^Tm	102	101	0,000	0,000
^173^Yb	104	102	0,000	0,000
^175^Lu	102	101	0,000	0,000
^205^Tl	120	115	0,000	0,000
^208^Pb	109	105	0,000	0,000
^209^Bi	108	103	0,000	0,000
^232^Th	101	100	0,006	0,021

**Table:** (a)

Discriminant function	Eigenvalue	Relative percentage	Canonical correlation
1	5.71366	73.38	0.92252
2	0.994461	12.77	0.70612
3	0.711944	9.14	0.64488
4	0.366289	4.7	0.51777

**Table:** (b)

Functions derived	Wilks *λ*	Chi-Square	DF	*P* value
1	0.0319289	265.2069	76	0.0000
2	0.2143590	118.5878	54	0.0000
3	0.4275310	65.4290	34	0.0010
4	0.7319100	24.0316	16	0.0888

**Table 4:** Concentration of elements (mg/kg) in olive oil samples analysed by ICP-MS.

Sample code	Harvesting mode	Water regime	Li	B	Na	Mg	Al	K	Ca	Sc	Cr	Mn	Fe	Co	Ni	Cu	Zn	Sr	Mo	Ba	La
			mg/kg	mg/kg	mg/kg	mg/kg	mg/kg	mg/kg	mg/kg	mg/kg	mg/kg	mg/kg	mg/kg	mg/kg	mg/kg	mg/kg	mg/kg	mg/kg	mg/kg	mg/kg	mg/kg
**K150**	**From the tree**	**TWW (alternate)**	0,080	0,000	0,202	56,586	16,332	0,000	0,869	0,011	0,152	0,058	3,775	0,060	0,050	0,006	133,349	0,014	0,049	0,185	0,074
**K173**	**From the tree**	**TWW (continuous)**	0,077	0,000	0,303	57,543	16,462	0,000	1,257	0,013	0,126	0,059	3,684	0,059	0,041	0,016	134,715	0,014	0,068	0,179	0,075
**K640**	**From the tree**	**CW**	0,079	0,053	0,537	53,690	14,701	0,431	1,330	0,013	0,129	0,331	3,578	0,059	0,038	0,061	131,816	0,020	0,056	0,177	0,071
**K661**	**From the tree**	**not irrigated**	0,079	0,000	4,654	50,292	14,402	3,953	1,753	0,012	0,107	0,102	3,882	0,059	0,037	0,076	122,980	0,013	0,038	0,178	0,071
**K151**	**First fall**	**TWW (alternate)**	0,077	0,079	0,429	59,414	17,790	0,000	1,461	0,013	0,143	0,072	4,229	0,063	0,045	0,008	134,205	0,015	0,044	0,191	0,063
**K174**	**First fall**	**TWW (continuous)**	0,078	0,155	0,738	55,682	17,367	0,000	1,582	0,014	0,139	0,084	4,804	0,062	0,029	0,019	135,066	0,037	0,041	0,185	0,074
**K644**	**First fall**	**CW**	0,079	0,000	5,791	31,538	8,973	0,000	0,990	0,018	0,112	0,061	4,062	0,060	0,072	0,064	141,022	0,016	0,036	0,176	0,077
**K664**	**First fall**	**not irrigated**	0,084	0,088	0,143	55,673	16,160	0,000	0,214	0,013	0,127	0,061	4,071	0,058	0,045	0,079	68,014	0,010	0,040	0,175	0,073
**K152**	**Second fall**	**TWW (alternate)**	0,080	0,094	1,110	57,455	16,879	5,467	0,000	0,017	0,133	0,062	4,136	0,063	0,035	0,003	135,414	0,011	0,048	0,183	0,076
**K175**	**Second fall**	**TWW (continuous)**	0,077	0,000	3,568	59,915	16,947	0,000	1,287	0,014	0,141	0,061	3,876	0,060	0,048	0,022	132,173	0,016	0,041	0,187	0,072
**K648**	**Second fall**	**CW**	0,029	0,000	3,898	61,493	17,427	0,000	3,000	0,018	0,121	0,063	3,689	0,060	0,034	0,067	134,367	0,016	0,047	0,188	0,072
**K668**	**Second fall**	**not irrigated**	0,073	0,022	4,089	59,968	17,397	51,288	4,461	0,009	0,160	0,072	3,883	0,060	0,065	0,082	134,062	0,019	0,045	0,188	0,072
**K153**	**Third fall**	**TWW (alternate)**	0,022	0,083	3,943	60,040	17,003	24,273	3,674	0,016	0,128	0,068	4,228	0,061	0,062	0,010	126,257	0,019	0,042	0,184	0,076
**K176**	**Third fall**	**TWW (continuous)**	0,053	0,327	16,586	61,289	21,159	15,623	3,409	0,015	0,114	0,067	4,349	0,086	0,059	0,025	142,977	0,075	0,157	0,204	0,078
**K652**	**Third fall**	**CW**	0,021	0,167	3,793	57,979	16,859	0,000	4,075	0,009	0,167	0,066	3,630	0,061	0,034	0,070	162,894	0,019	0,042	0,184	0,054
**K672**	**Third fall**	**Not irrigated**	0,022	0,129	1,555	63,346	25,625	0,000	3,344	0,010	0,160	0,076	4,411	0,076	0,061	0,085	158,817	0,042	0,051	0,201	0,076
**K154**	**Forth fall**	**TWW (alternate)**	0,080	0,143	4,629	57,887	17,235	53,418	2,551	0,038	0,137	0,072	4,144	0,059	0,036	0,031	124,821	0,023	0,033	0,190	0,072
**K187**	**Forth fall**	**TWW (continuous)**	0,022	0,098	7,846	61,749	17,337	98,075	1,536	0,021	0,146	0,073	4,104	0,062	0,044	0,028	127,282	0,045	0,048	0,194	0,080
**K656**	**Forth fall**	**CW**	0,016	0,090	2,642	58,431	18,083	0,000	1,890	0,021	0,159	0,066	4,146	0,060	0,051	0,073	95,394	0,015	0,042	0,185	0,070
**K677**	**Forth fall**	**Not irrigated**	0,075	0,088	0,523	61,106	18,244	0,000	4,268	0,050	0,160	0,066	17,596	0,068	0,046	0,088	129,939	0,018	0,043	0,187	0,073
